# Effects of Induced Malocclusion on Vertebral Alignment in Rats: A Controlled Study by CBCTs

**DOI:** 10.3390/ani11102808

**Published:** 2021-09-27

**Authors:** Michele D’Attilio, Gianfranco Cesaretti, Paolo Viganò, Karol Alí Apaza Alccayhuaman, Daniele Botticelli, Erick Ricardo Silva, Samuel Porfirio Xavier

**Affiliations:** 1 Department of Innovative Technology in Medicine and Dentistry, University of Chieti-Pescara, 66100 Chieti, Italy; 2 Private Practice, Castelfidardo, 60022 Ancona, Italy; giancesa55@gmail.com; 3 ARDEC Academy, 47923 Rimini, Italy; paolovigano63@gmail.com (P.V.); caroline7_k@hotmail.com (K.A.A.A.); daniele.botticelli@gmail.com (D.B.); 4 Private Practice, Settimo Milanese, 20019 Milano, Italy; 5 Depto CTBMF e Periodontia FORP-USP, Faculty of Ribeirão Preto (SP), 14000-000 Ribeirão Preto, Brazil; erickricardo.rp@gmail.com (E.R.S.); samuelpxavier@yahoo.com.br (S.P.X.)

**Keywords:** occlusion, vertebral alignment, occlusal splint, radiography, posture, rats

## Abstract

**Simple Summary:**

Nowadays, there is insufficient available evidence to validate a correlation between dental occlusion disorders and spinal curvature, and therefore, more studies are necessary to clarify how and to what extent the modifications of the occlusion might affect vertebral spine biomechanics. The following paper is a controlled study on rats that aims to induce a malocclusion in rats’ mouths by a bite-raising on the upper molars and then to check the alteration of vertebral alignment over time by a CBCT analysis. Alteration of vertebral alignment in rats with induced malocclusion could support the hypothesis of a correlation between occlusal contact and body posture and open the way for further studies on mandibular repositioning and posture control in humans.

**Abstract:**

This study aimed to evaluate with CBCTs the alteration of vertebral alignment over time of induced malocclusion in rats. Crown pads increasing the vertical dimension of 0.5 mm were applied on the upper molars at one randomly selected side of the maxilla in rats (premature contact side) while the opposite side was left untreated (control side). Four groups were organized, ten animals each. In groups A, B, and C, the crowns were applied at time 0 (t-0). In group A, the crowns were removed after 2 weeks (t-2w) and euthanized after two more weeks, while in groups B and C, the animals were euthanized after 2 and 4 weeks (t-4w), respectively. No premature contacts were applied in group D. CBCTs were taken at t-0 in all animals before applying the crowns, at t-2w in group A before removing the crowns, and in all groups before the euthanasia. The changes in the iliac crest angle (ICA) that formed between the superior external margin of the iliac crest and the vertebral spine were evaluated. In groups A and B, after 2 weeks, the changes in ICA were statistically significant at *p* = 0.028 and *p* = 0.042, respectively. In group C, and in the control group D, the changes of ICA were not statistically significant (*p* = 0.058 and *p* = 0.414, respectively). In conclusion, the incease in monolateral occlusion in the molar region yielded a rotation of the lumbo-sacral segment towards the same side of the occlusal bite-raising.

## 1. Introduction

The nerve conduction and anatomy of the masticatory system have an intimate relationship with the vertebrae column. There is an intimate relationship between the trigeminal nerves and both sensation and movement of the neck. The nucleus of the caudal spinal trigeminal nucleus interlocks with the nerves of the cervical cord, and the trigeminal nerves participate in the formation of the nerves of the cervical cord at the C1 and C2 regions. Kerr [1] injected bradykinin into the masseter muscle of cats and observed the reaction of muscle spindle of trapezius at C3 and C4, which demonstrated that the chemical change in the masseter nerve periphery had an effect on the afferent impulsion of muscle spindle of the back and neck muscles. Due to the attachment of muscles and ligaments, the cranium and mandible form an organic unction system. The temporalis, sternocleidomastoid, and trapezius participate in maintaining the rest position and movement of the mandible [2,3]. Experimental occlusion interference could cause the joint movement of the upper cervical vertebrae and sacral vertebrae to be abnormal [4]. In orthodontic treatment, increasing the occlusal vertical dimensions (OVD) for a long time could lead to position abnormality of the cervical vertebrae [5]. These studies implied that the masticatory system, head, neck and vertebrae had an intimate relationship in terms of nerves and anatomy. In recent years, studies have investigated the influence of craniofacial parameters using three-dimensional measurements of the dorsal profile, trunk inclination and pelvic tilt and rotation by means of rasterstereography [6,7]. Patients with malocclusion and bruxism had a larger incidence of head and neck pain, and the asymmetry of dental occlusion could cause chronic pain in the neck [8,9]. The finite element stress analysis demonstrated that the obliquity of the occlusal plane could lead to mandible stress transferring to cervical vertebras, inducing compensatory change [10,11]. Animal experiments revealed that rats with a unilateral increased OVD might have experimentally induced scoliosis [12], but some other studies have demonstrated that there was no detectable correlation between dental occlusion and body posture [13]. However, no sufficient evidence is available to validate a correlation between dental occlusion disorders and spinal curvature [12]. Owing to the difficulties in carrying out clinical trials on humans, animal studies might provide clarifications about the matter discussed above.

Joint and tissue mechanoreceptors play an important role in static upright regulation. These receptors are affected by the posture, which can change the input into the central nervous system [14]. Animal studies can be very useful to evaluate the effect of dental occlusion on the vertebral spine. Nevertheless, the interpretation of the results should take into consideration the differences between quadrupedalism and bipedalism [15]. Moreover, previous animal studies have used a 2D radiographic evaluation, but 3D assessments are still missing, such as on cone bean computed tomographies (CBCTs). Hence, the aim of the present study was to evaluate with CBCTs the alteration of vertebral alignment over time of induced malocclusion in rats.

## 2. Materials and Methods

### 2.1. Ethical Statement

The protocol of the experiment was approved by the Ethical Committee of the Faculty of Dentistry of Ribeirão Preto of São Paulo University, Brazil (2017.1.583.58.6). The regulations of Brazilian animal care were accurately followed. The ARRIVE checklist for animal studies was applied.

### 2.2. Study Design

In this experimental study, a malocclusion was induced with crowns increasing the vertical dimension applied on the upper molars at one randomly selected side of the maxilla in rats (premature contact side) while the opposite side was left untreated (control side). Four groups, each composed of 10 animals, were formed. In groups A, B, and C, the crowns were applied at time 0 (t0). In group A, the crowns were removed after 2 weeks (t-2w), and the animals were sacrificed after 4 weeks (t-4w). In group B, the animals were sacrificed after 2 weeks. In group C, the animals were sacrificed after 4 weeks with no other interventions. Group D was used as a control and no crowns were applied or sacrificed after 4 weeks. CBCTs were taken at t-0 in all animals before applying the crowns, at t-2w in group A before removing the crowns, and in all groups before the euthanasia.

### 2.3. Experimental Animals

The rats in the study were provided by the Central Bioterium of the University of Sao Paulo. Forty 20–22-week-old, sexually mature male Sprague Dawley rats (average weight at t-0 470 ± 21 g) with normal occlusion and vertebral alignment were included in the study.

### 2.4. Sample Size

The evaluation of the sample size was based on the data from another study performed by the first author [12]. A type I error of 0.05 and a power of 0.8 were applied for matched pairs, and a sample size of 9 was obtained for each group.

### 2.5. Randomization and Allocation Concealment

**Figure 1:**
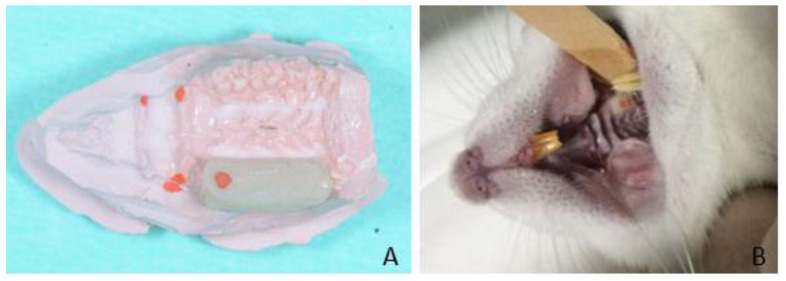
(**A**) Cast model of the upper jaw of a rat with the crown pad in position. (**B**) Crown pad fixed to the maxillary molars. An occlusal control was performed to verify the premature contacts (red points).

To perform the randomization, the website www.randomization.com was used by one author (DB) not involved in the prosthetic procedures. Sealed opaque envelopes reporting the side of the maxilla in which to apply the crowns were prepared and opened at the prosthetic sessions. Anesthesia was carried out using a mixture of ketamine (90 mg/kg, i.p., Dopaser, Sespo Indústria e Comércio LTDA, Paulínia, Sao Paulo, Brazil) and xylazine (10 mg/kg, i.p., Anasedan, Sespo Indústria e Comércio LTDA, Paulínia, Sao Paulo, Brazil). As the first step of the procedure, an impression on maxillary full dentition was taken in one animal using an individual tray and a silicone impression material (President, Coltene/Whaledent AG, Altstaetten, Switzerland) and was poured with die stone (Elite Master, Zhermack spa, BAdia Polesine, RO, Italy). Based on this cast, preformed resin devices (Signum composite, Kulzer, Milan, Italy) were fabricated with a standardized thickness of 0.5 mm (Figure 1A) according to previous studies [12,16]. These devices were bonded to the upper posterior teeth by dual curing composite resin (Filtek Supreme Flowable, 3M Italia, Pontello, MI, Italy) at time 0 (t-0) in the rats of groups A, B, and C (Figure 1B).

### 2.6. Euthanasia

All rats were euthanized with intraperitoneal injections of 10 mg/mL lidocaine (0.7 mg/kg body weight) associated with 2.5% sodium thiopental (Thiopentax; Cristália Produtos Químicos Farmacêuticos Ltd.a., Paulínia, SP, Brazil; 150 mg/kg body weight) after each experimental period proposed.

### 2.7. Housing and Husbandry

The rats were maintained in individual cages in a room at 25 °C with a 12–24 h light/dark cycle with free access to food and water at the animal facilities of the Faculty of Dentistry of Ribeirão Preto, University of São Paulo (Brazil). Professionals provided for the care of the animals, monitoring wounds and biological functions and controlling pain and infection using appropriate drugs prior to, during and after the experiment.

### 2.8. CBCTs Procedures

**Figure 2:**
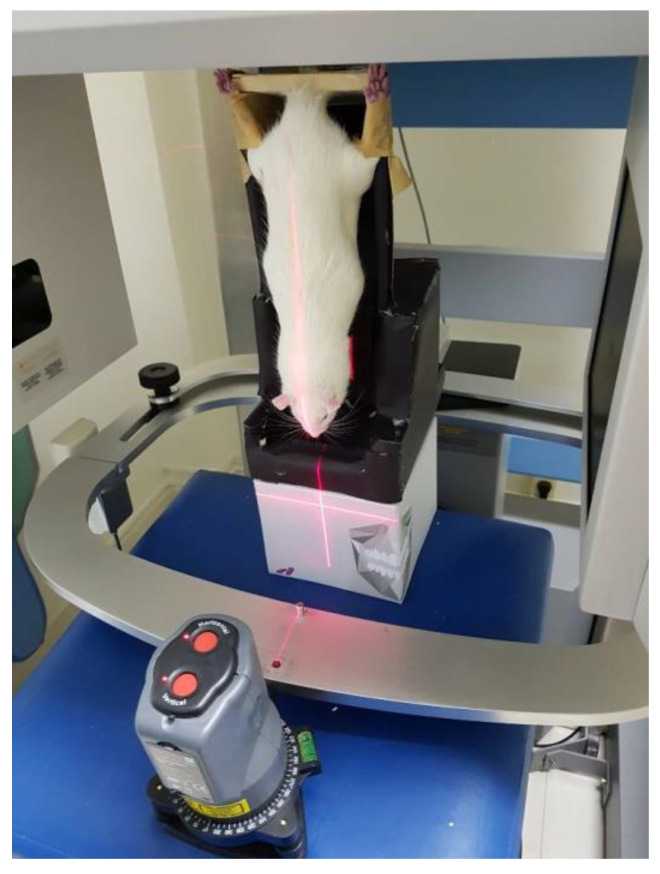
A self-made box made of expanded polystyrene was used and placed in the CBCT equipment. The legs of the animals were fixed to the box with adhesive plaster tape, and the position of the body was adjusted using a laser pointer that provided the control of both the vertical and the horizonal planes.

**Figure 3:**
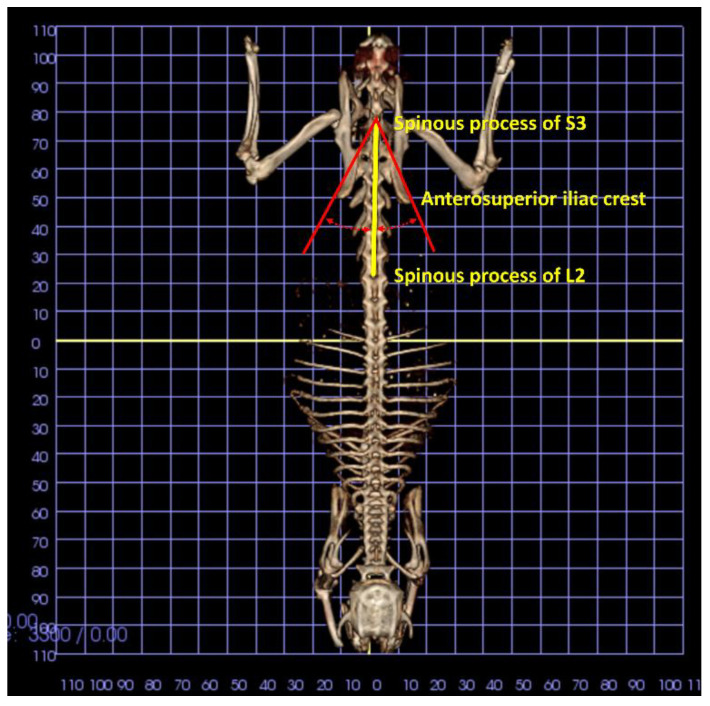
In the dorsal view, the spinous processes of S3 and of L2 were identified and connected by a line (in yellow). Moreover, the superior external margin of the iliac crest was identified and connected by lines to S3 bilaterally (in red). The angle (iliac crest angle; ICA) formed between the two lines was measured both in the right (right iliac crest angle) and in the left side (left iliac crest angle).

The animals were sedated before the CBCTs were taken. A self-made box made of expanded polystyrene was used (Figure 2). The legs of the animals were fixed to the box with an adhesive plaster tape, and the position of the body was adjusted using a laser pointer (Livello Laser Cross Beam Meister Kreuzlinienlaser MKL 100-Laseklasse 2-Meister Werkzeuge, Oberkamper StraBe 37–39 D 42349 Wuppertal) that provided the control of both the vertical and horizontal planes (Figure 2). For the 3D analysis, the volume rendering was opened with the program In vivo 5.0 (Anatomage, San José, CA, USA), and the measurements were processed using a superimposed grid for the 3D positioning of the sample. In the dorsal view, the spinous processes of S3 and L2 were identified and connected by a line (Figure 3). Moreover, the superior external margin of the iliac crest was identified and connected bilaterally by a line to S3. The angle (iliac crest angle; ICA) formed between the two lines was measured both in the right (right iliac crest angle) and in the left side (left iliac crest angle).

### 2.9. Calibration for Histometric Evaluations

All assessments were carried out by a well-trained examiner (K.A.A.A.). Measures were taken twice, with an interval of one week between the two measurements, and mean values were used. The intra-rater agreement in the measurements was K > 0.90.

### 2.10. Experimental Outcomes and Statistical Analyses

The primary outcome was the difference in iliac crest angles measured at t-0 in relation to those taken in the subsequent periods. The Wilcoxon rank sum test using the software IBM SPSS Statistics (IBM Inc., Chicago, IL, USA) was applied for statistical evaluation.

## 3. Results

### 3.1. General Information

The animals increased weight over time, by about 20–30 g after 2 weeks and about 160 g after 4 weeks. The differences in weight between groups were not statistically significant for any group in any period evaluated.

### 3.2. Group A 

**Figure 4:**
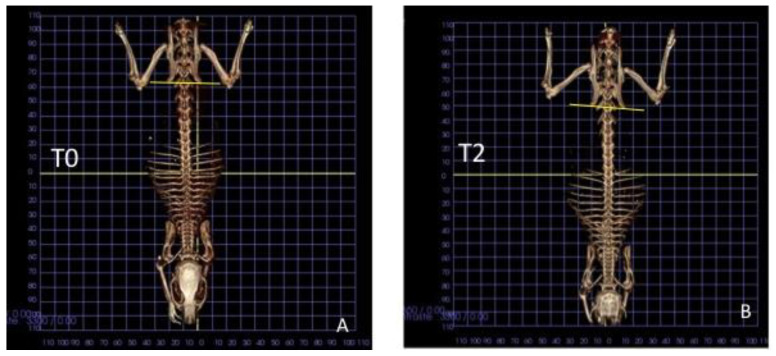
CBCT images taken in a rat of group A after (**A**) and before the fixation of the crown pad; (**B**) situation after 2 weeks of unilateral premature contact. Note the deviation of the two iliac crest angles.

The iliac crest angle (ICA) at t-0 was 21.4 ± 1.9° and 21.7 ± 1.0° (*p* = 0.477) in the premature contact and control sides, respectively (Table 1). Two weeks after that, one rat was found without the crowns, and it was excluded from analysis. Changes in the angles were seen in both groups, being 3.2 ± 1.2° and 1.3 ± 1.0° in the premature contact and control sides, respectively (Figure 4A,B). The difference was statistically significant (*p* = 0.028). The crowns were removed, and after two weeks the changes of ICA in relation to the baseline decreased to 1.4 ± 1.6° and 0.4 ± 1.1° (*p* = 0.192).

### 3.3. Group B 

The iliac crest angle at t-0 was 22.2 ± 1.2° and 22.2 ± 1.2° (*p* = 0.108) in the premature contact and control side, respectively (Table 2). One animal died after the surgery, and two were found without the crowns after 2 weeks and were excluded from the analysis. In the CBCT analyses after 2 weeks, the difference in the ICA was 2.0 ± 1.2° at the premature contact and 0.9 ± 0.7° at the control side. The difference was statistically significant (*p* = 0.042).

### 3.4. Group C 

The iliac crest angle at t-0 was 22.2 ± 1.5° and 22.9 ± 1.3° (*p* = 0.108) in the premature contact and opposite sides, respectively (Table 3). After 4 weeks, one rat was found without the crown and was excluded from analyses. in the CBCTs, the difference for ICA was 1.5 ± 0.5° at the premature contact and 0.6 ± 1.1° at the control side. The difference did not reach statistical significance (*p* = 0.058).

### 3.5. Group D 

The ICA at t-0 was 21.6 ± 0.8° and 22.5 ± 1.4° (*p* = 0.283) (Table 4). After 4 weeks of no treatments, the differences between right and left sides were 1.0 ± 0.8° and 0.7 ± 1.3°, respectively (*p* = 0.414).

### 3.6. Differences among Groups

No statistically significant differences were seen among groups after either 2 weeks (A vs. B) or 4 weeks (A, vs. C and vs. D)

## 4. Discussion

Several studies dealing with the relationship between dental occlusion and body posture have been published in the worldwide literature. The main limit of these studies was the extreme heterogeneity of material and methods employed that made any comparisons very difficult. Nevertheless, two of these studies [12,17] applied the same study design that was used in the present experiment. However, instead of a 2D radiographic assessment, a tomographic 3D evaluation has been used.

The muscle adaptation to the change in the mandible’s position might influence vertebrae alignment and vice versa. This study changed the occlusal pattern of rats with a crown pad bonded on the first right molar, in one group and on the first left molar in the other group. The expected results were that the muscles of hoisting vertebrae would maintain normal head posture and the balance of body, which might cause rats to lose the centric position and develop occlusal deviation; due to the height of the splints, the rats needed to adjust their muscles in head and neck, even increasing OVD unilaterally for a week. In the present study, rats developed a deviation at T10 vertebrae alignment to achieve an adaptive change from cervical vertebrae and thoracic vertebrae to lumber vertebrae.

The results from the present study showed significant differences in the vertebral alignment, measured by the iliac crest angle (ICA) when dental occlusion was altered. After either 2 (group A,B) or 4 weeks (group C) from the inclusion of an experimental malocclusion by means of the application of a crown pad for a unilateral bite-raising, there was a significant modification of ICA in both the right and the left sides. Moreover, the premature contact side showed higher values of the ICA when compared with the control side: this suggests the hypothesis that a unilateral increase in the vertical dimension could induce a change In the lumbar–sacral vertebrae alignment, represented by a rotation of the iliac crests plane in the same side of the bite-raising.

On the other hand, the control group (D) did not show significant modifications in CBCT analysis of vertebral alignment after 4 weeks. However, the angle increased between the two periods, showing that the angle was positively influenced by the increased weight of the rats. This, in turn, means that the increased dimensions of the animals might have also influenced the increased angle in the other groups. Nevertheless, the difference between premature and control sides within groups was 1.9° in group A (*p* = 0.028) and 0.9° in group C (*p* = 0.058). Moreover, by comparing the sides with premature contacts in groups A and C to the mean values of the control group D after 4 weeks, a difference in the angle of 0.6–0.7° was shown, even though statistically significant differences were not achieved.

It was interesting to notice that, in group A, crown pad removal after 2 weeks since the installation resulted, after 2 more weeks, in a reduction in the changes in ICA compared to those found after t-2w, even though the baseline values were not yet completely restored. This confirms the results observed by another similar experiment [11] that showed that the vertebral spine gained a realignment when the dental occlusion was stabilized.

In group C, the follow-up time of the unilateral bite-raising up to 4 weeks (group C) did not determine a further increase in column rotation compared to that observed after 2 weeks in group A. It might be speculated that this outcome could be associated with an abrasion of the crown over time that decreased the malocclusion, or it may be associated with an adaptation of the column. Indeed, it was shown that the temporomandibular joint mechanoreceptors will adapt over time to increase in the occlusal vertical dimension [18].

Modifications in height of dental occlusion might result in changes in masticatory muscles activity. These muscles connect the mandible to the vertebral spine and might affect the vertebral spine curvatures at the level of cervical and thoracic regions [17]. As the body presents a tendency of adaptation to the new condition, the reduction in the unilateral over-occlusion due to the abrasion of the crown pads, the cervical and thoracic spines tends to realign, reducing the curvature of the lumbar spine. This agrees with the results reported in other rat experiments [12,17] in which unilateral increase in OVD might induce scoliosis. However, these two studies did not report data on an association between the side of OVD and the direction in which the vertebral spine is displaced. In a previously reported study, [12] the increase in OVD determined a mandible lateral shift and a monolateral posterior cross-bite. This mandible rotation likely did not allow a direct relationship to be determined between mandible and column shift sides. On the contrary, the present study was designed to evaluate the simple effect of OVD augmentation on vertebral alignment without introducing any mandible rotation. Therefore, the results from the present study suggest that the unilateral increase in OVD could produce an inclination of the occlusal plane on the frontal plane so that the vertebral spine rotates towards the same side.

Furthermore, the data discussed in the present report have been assessed with CBCTs, while other animal and human studies evaluated the correlation between occlusion and posture by X-ray 2D evaluation. It might be suggested that the measurements performed in the present study based on a 3D evaluation could be considered more reliable than those based on 2D analysis.

In the present study, an increased occlusal height in one side of the jaws produced malocclusion. However, a malocclusion corrected bilaterally with increased vertical dimensions and a deep overbite improved the control of jaw movements during chewing [19].

As a limitation of the present study, it is worth mentioning the material used to produce the crown pads that did not avoid abrasion or fractures beyond the 2-week period of observation. This condition led to a reduced sample and a loss of power. The standardization of the positioning of the animal for the scanning was complex. Horizontal positioning of the animal would be preferable if possible. In the case of a more detailed analysis of smaller structures such as TMJ and crowns, the definition of the images was not ideal. A microscopic evaluation of the residual thickness of the crowns would have added further information about abrasion and loss of height of the premature contact. Moreover, before human experiments, future experiments should be performed in animal models not requiring euthanasia and with bipedal vertebral column structure. This animal model could be monkeys. Finally, clinical studies should collect records with 3D rather than 2D technology.

## 5. Conclusions

In conclusion, the increase in monolateral occlusion in the molar region yielded a rotation of the lumbo-sacral segment towards the same side of the occlusal bite-raising.

## Figures and Tables

**Table 1:** Group A. Iliac spine angles expressed in grades at baseline (t-0), after 2 weeks (t-2w), at the removal of the crowns, and after 4 weeks (t-4w) from baseline. The CBCT measurements were performed at the side of the premature contact and at the opposite side.

	Premature Contact Side	Opposite Side	*p*-Value
t-0	21.4 ± 1.9	21.7 ± 1.0	0.477
t-2w	24.6 ± 2.4	23.0 ± 1.2	0.066
t-4w	22.7 ± 1.6	22.1 ± 0.8	0.260
Difference t-2w and t-0	3.2 ± 1.2 *	1.3 ± 1.0 *	0.028
Difference t-4w and t-0	1.4 ± 1.6	0.4 ± 1.1	0.192

* Statistically significant result.

**Table 2:** Group B. Iliac spine angles expressed in grades at baseline (t-0) and after 2 weeks from the delivery of the crowns (t-2w). The CBCTs measurements were performed at the side of the premature contact and at the opposite side.

	Premature Contact Side	Opposite Side	*p*-Value
t-0	22.2 ± 1.2	22.2 ± 1.2	0.866
t-2w	24.1 ± 1.8	23.2 ± 0.9	0.204
Difference t-2w and t-0	2.0 ± 1.2 *	0.9 ± 0.7 *	0.042

* Statistically significant result.

**Table 3:** Group C. Iliac spine angles expressed in grades at baseline (t-0) and after 4 weeks since the delivering of the crowns (t-4w). The CBCTs measurements were performed at the side of the premature contact and at the opposite side.

	Premature Contact Side	Opposite Side	*p*-Value
t-0	22.2 ± 1.5	22.9 ± 1.3	0.108
t-4w	23.7 ± 1.6	23.5 ± 1.5	0.155
Difference t-4w and t-0	1.5 ± 0.5	0.6 ± 1.1	0.058

**Table 4:** Group D. Iliac spine angles expressed in grades at baseline (t-0) and after 4 weeks with no treatment. The CBCT measurements were performed on the right and left sides of the animals.

	Right	Left	*p*-Value
t-0	21.6 ± 0.8	22.5 ± 1.4	0.283
t-4w	22.6 ± 1.1	23.1 ± 1.1	0.153
Difference t-4w and t-0	1.0 ± 0.8	0.7 ± 1.3	0.414

## Data Availability

The data presented in this study are available on request from the corresponding author.
